# Distribution of HCV Genotypes in the Populations of Inmates in Polish Prison Potulice and Patients Hospitalised in Bydgoszcz

**DOI:** 10.5812/hepatmon.14559

**Published:** 2014-05-03

**Authors:** Malgorzata Tyczyno, Waldemar Halota, Wojciech Nowak, Małgorzata Pawlowska

**Affiliations:** 1 Department of Infectious Diseases and Hepatology, Nicolaus Copernicus University, Bydgoszcz, Poland

**Keywords:** Hepatitis C, HCV, Prison

## Abstract

**Background:**

According to many studies, one of the social groups with high rate of HCV infections are prisoners.

**Objectives:**

The aim of the study was to determine and compare the genotypes distribution among prisoners and patients of hospital.

**Patients and Methods:**

HCV genotypes among prisoners (281 inmates) and patients of hospital (1415 patients) were determined in years 2002-2012. HCV genotypes were determined in 2002-2005 with INNO-LiPA HCV II test (Innogenetics, Gent, Belgium) and since 2006 with LINEAR ARRAY assay (Roche, Mannheim, Germany), after isolation and amplification of the material with COBAS AMPLICOR v 2.0 (Roche, Mannheim, Germany).

**Results:**

The most frequent HCV genotype among inmates was genotype 3, which was detected in169 of 281 patients (60.1%). Most frequent genotype among hospitalized was genotype 1, which was found in 1127 cases (79.6%). Comparing the results of prisoners with a group of patients with HIV/HCV co-infection gave similar results. In both groups most frequent was genotype 3 (respectively 60.1 and 45.5%). However, most prisoners in this study (96%) were HIV-negative.

**Conclusions:**

The current study shows that the predominant HCV genotype among inmates from prison in Potulice is genotype 3.

## 1. Background

According to the estimations of the World Health Organization about 3% of people (170-200 million) worldwide are infected with hepatitis C virus (HCV). The prevalence of HCV infection varies throughout the world. The smallest percentage, about 1%, reported in north-western Europe, North America and Australia. The highest number of infections reported in Egypt (about 22%).

In Europe the smallest amount of HCV infection, approximately 0.5% is reported in the northern part. It is estimated that 1% of infected persons live in the north-western part of the continent, 2% in the east and as many as 4.6% in the Mediterranean. In Poland infected are approximately 1.5% of the population (1, 2). In the HCV genome were observed differences in the nucleotide sequence of the region encoding the nonstructural protein (NS5). On the basis of the similarity of nucleotide sequence genotypes, subtypes and quasispecies were separated. This similarity in the case of genotypes ranges between 65, 7 and 68,9%. Whereas in subtypes the range is from 76,9 to 80,1%, quasispecies it is 90,8-99% of similarity of nucleotide sequence (3). There are six main genotypes of HCV. Their presence in the world is diversified geographically. Genotypes 1 and 2 are the most common in Europe, the United States of America and Japan (4). Genotype 3 is mainly in Europe and the United States of America, particularly among intravenous drug users, as well as in India and Pakistan (2, 5, 6). Genotype 4 is the most common in the Middle East and Africa, 5 in South Africa and 6 in Asia (7, 8). In Vietnam genotypes 7, 8 and 9 were identified; in Indonesia, 10 and 11 because their presence is more local, the standardized nomenclature classified them into one group - genotype 6 (9). It has been observed that not only the geographical distribution may affect the type of genotype. Age, route of infection, and co-infections may also have influence (10-12).

Regardless of the geographic variable, the incidence of HCV infection may vary in certain social groups. One such group are the prisoners. Within this group HCV infection was found in 20-47% of inmates (13-15). In these patients, the HCV infection occurs mainly as a result of intravenous drug use, unprotected sex and tattooing or piercing. Wherein it is believed that the HCV infection in these patients group may occur both before and during incarceration (16, 17).

## 2. Objectives

The aim of the study was to determine and compare the genotypes distribution among prisoners and patients hospitalised in Bydgoszcz. Because according to many studies, one of the social groups with high rate of HCV infections are prisoners, and no study has been done in order to determine HCV genotypes in group of inmates.

## 3. Patients and Methods

The study involved 281 inmates from prison in Potulice (group I) and 1415 adult persons from Department of Infectious Diseases in Bydgoszcz (group II). All diagnosed in hospital in Bydgoszcz in years 2002-2012 due to HCV infection. Among patients from Bydgoszcz there was an isolated group of 132 patients with HIV/HCVco-infection (group III). HCV genotypes were determined in 2002-2005 with INNO-LiPA HCV II test (Innogenetics, Gent, Belgium) and since 2006 with LINEAR ARRAY assay (Roche, Mannheim, Germany), after isolation and amplification of the material with COBAS AMPLICOR v 2.0 (Roche, Mannheim, Germany).

## 4. Results

Comparison of the results showed significant differences in the prevalence of HCV genotypes in different groups (Table 1).

**Table 1.** HCV Genotype in Group Inmates (I) and Hospitalized (II) ^a^

Group	1	2	3	4
**Inmates (I), n = 281**	94 (33,5)		169 (60,1)	18 (6,4)
**Hospitalised (II), n = 1415**	1127 (79,6)	1 (0,1)	161 (11,4)	126 (8,9)

^a^ Data are presented in No. %.

Among group I prevailed genotype 3, which was detected in 169 of 281 patients (60.1%). Genotype 1 was present in 33.5% and 4 in 6.4% of inmates. In group II observation showed more frequently genotype 1, which was found in 1127 cases (79.6%). Genotypes 3 and 4 were similar, they were detected respectively in 161 and126 of 1415 adult patients. Comparison of the results from patients within group I and III gave similar results (Table 2). In both groups genotype 3 (respectively 60.1 and 45.5%) was most frequent. Genotype 1 has performed with nearly identical frequencies: 33.5 and 33.3%. Genotype 4 was found in 6.4% of group II and 21.2% of group III.

**Table 2.** HCV Genotype in Group Inmates (I) and HIV/HCV (III) ^a^

Group	1	2	3
**Inmates (I), n = 281**	94 (33,5)	169 (60,1)	18 (6,4)
**HIV/HCV (III), n = 132**	44 (33,3)	60 (45,5)	28 (21,2)

^a^ Data are presented in No. %.

## 5. Discussion

HCV genotype 3 was dominant, detected in 60% of patients diagnosed in Potulice. It seems that these results may be related to a tightness of the group. This may be the reason in the distribution differences of HCV genotypes obtained during the study of their occurrence among prisoners in the world. In Spain and Canada HCV genotype1 and 3 occurred at similar rates (respectively about 50 and 30%). Among the prisoners in Spain the presence of genotype 4 in 17% of patients was also found. This type has not been detected among Canadian prisoners, instead of 12% of genotype 2 (18, 19). The Brazilian researchers have found the presence of genotype 1 in 91% of prisoners (20). Research has indicated that the most common type of HCV with patients from Bydgoszcz is genotype 1, occurring at less than 80% of the respondents. These results coincide with those obtained in 2006, when the genotype 1 was found in over 80% of adult patients in Bydgoszcz (21). Similarly Chlabicz et al. demonstrated the predominance of genotype 1 in the study group, reported it in 57% of patients (22). This is in line with other European reports, where genotype 1 was detected in 68-90% of patients (23, 24). The incidence of HCV genotypes among inmates in Potulice was similar to that which was found among a group of patients with HIV/HCV co-infection diagnosed in Bydgoszcz. In both groups genotype 3 HCV is the most common. However, among patients in Potulice only 4% of them have HIV/HCV co-infection. Similar results of HCV genotypes distribution among HIV/HCV co-infected patients were also received by Grabczewska and Babik, Holodniy (25, 26), they demonstrated the predominance of genotype 3 in 50-60% of cases. Karakulova and Power demonstrated the predominance of genotype 1 among HIV/HCV co-infected patients (27, 28). Similarly to the prisoners these results may be related to the tightness of the group.

Likewise, such a predominance of HCV genotype 3 was demonstrated also in HCV monoinfected patients. These patients indicated intravenous drug use as the likely source of HCV infection (12, 22). During the examination, 125 of the 281 patients from Potulice indicated intravenous drug use as the probable source of HCV infection. Of these, 56% had genotype 3 HCV; genotype 1 was present in 25.5% and4 in 18.5% of inmates in Potulice.

The above results require further studies in larger patient populations.
